# Antibody detection against Kunitz-type protein in *Fasciola hepatica* experimentally infected sheep using enzyme-linked immunosorbent assay (ELISA)

**DOI:** 10.1080/23144599.2023.2273678

**Published:** 2023-11-14

**Authors:** María Ahumada, Agustina Godino, Lorena Guasconi, Carla Deheza, Marilla Amaranto, Cesar Iván Pruzzo, Gabriel Vitulli-Moya, Laura Chiapello, María Elena Carrizo, José Luis Barra, Laura Cervi

**Affiliations:** a Departamento de Bioquímica Clínica, Facultad de Ciencias Químicas, Universidad Nacional de Córdoba, Córdoba, Argentina; b Centro de Investigaciones en Bioquímica Clínica e Inmunología (CIBICI), Consejo Nacional de Investigaciones Científicas y Técnicas (CONICET), Córdoba, Argentina; c Facultad de Ciencias Agropecuarias, Universidad Católica de Córdoba, Córdoba, Argentina; d Instituto Nacional de Tecnología Agropecuaria (INTA) Estación Experimental Agropecuaria Manfredi, Córdoba, Argentina; e Departamento de Química Biológica Ranwel Caputto, Facultad de Ciencias Químicas, Universidad Nacional de Córdoba, Córdoba, Argentina; f Centro de Investigaciones en Química Biológica de Córdoba (CIQUIBIC), Córdoba, Argentina; g Departamento de Epizootiología y Salud Pública, Universidad Nacional de La Plata, La Plata, Argentina; h Centro de Diagnósticos e Investigación Veterinaria (CEDIVE), Universidad Nacional de La Plata, La Plata, Argentina

**Keywords:** Sheep, *Fasciola hepatica*, early diagnosis, Kunitz-type protein, antibody detection

## Abstract

Fasciolosis is a parasitic disease considered as emerging and neglected by the WHO. Sheep are highly susceptible to this disease, and affected flocks experience decreased productivity due to increased mortality, and the reduced quality of their products, such as wool and meat. To effectively control this disease, reliable and early diagnosis is essential for making decisions regarding antiparasitic application and/or the removal of affected animals. Currently, the diagnosis of *F. hepatica* in sheep relies on the detection of parasite eggs in faeces, a method that becomes reliable from week 10 post-infection. Consequently, there is a need for earlier diagnostic tools based on immune response. However, obtaining antigens for antibody detection has proven to be difficult and expensive. The aim of this study was to evaluate members of the Kunitz protein family of *F. hepatica* expressed in the form of a fusion protein in the serological diagnosis of *F. hepatica* in sheep. The performance of three recombinant *F. hepatica* Kunitz-type inhibitors (FhKT1.1, FhKT1.3, and FhKT4) was compared with a synthetic Kunitz-type peptide (sFhKT) in sera from sheep experimentally infected with *F. hepatica*, using an ELISA. Of these, FhKT1.1 showed the most promising diagnostic indicators, exhibiting high precision and low cross-reactivity, and thus potential for standardized production. The results of our study demonstrated that the application of FhKT1.1 is a valuable tool for early-stage diagnosis of *F. hepatica* in sheep. Such an early diagnosis can aid in implementing timely interventions and effectively managing the disease in sheep populations.

## 1. Introduction

Fasciolosis, caused by the helminth parasite *F. hepatica*, is a zoonosis with a worldwide distribution that is continuously expanding. This expansion is seen in the diversity of mammalian hosts infected, including donkeys, horses, alpacas, mules, and otters [1], as well as in the adaptation of its intermediate hosts (snails of the Lymnaeidae family) to a wide range of environments [2,3]. Sheep and cattle are the main hosts for fasciolosis worldwide, with a prevalence of above 80% in these ruminants in endemic areas [1,2,4]

The economic damage produced by fasciolosis in the livestock industry is challenging to estimate but is calculated to be around US$ 3 billion annually [4]. These losses are not solely attributed to the loss of livers in slaughterhouses but also result from reduced daily weight gain, and decreased milk and wool production in animals affected by this parasite.

The life cycle of this helminth comprises a series of stages, with the parasite taking 8–12 weeks to settle in the liver of mammals.

A simple method used for the diagnosis of parasitosis is the detection of eggs in the faeces of mammalian hosts; however, this is not viable in the early stages of infection and *F. hepatica* parasites start inducing a humoral immune response as early as two weeks post infection, depending on the host.

For decades, the methods for antibody detection against *F. hepatica* in serum used native, excreted-secreted, or tegumental antigens, making it difficult to obtain standardized antigen production [5]. However, recombinant antigens have been developed to avoid the disadvantages of the variation in quality and quantity of antigens used for *F. hepatica* diagnosis [6]. The sensitivity and specificity of techniques for antibody detection against *F. hepatica* in different species was reviewed by Alvarez Rojas et al. (2014). Cathepsin L1 (CL1) is one of the most frequently used antigens for *F. hepatica* diagnosis, due to its high expression in *Fasciola* species [7,8]. Despite the high specificity and sensitivity levels achieved when using recombinant cathepsin L1 (rCL1), its expression system may require the use of yeasts such as *Pichia pastoris*, which is a more expensive and difficult protein production system compared to other expression vectors such as *E. coli* [9]. Furthermore, rCL1 expression in *E.coli* occurs in the form of inclusion bodies, so the protein must be purified with denaturing agents and subsequently refolded [10]. Interestingly, the antibody response in naturally or experimentally infected cattle and sheep varies qualitatively. Additionally, not all naturally infected animals display detectable antibody responses when tested with either native or recombinant CL1 antigens. This underscores the need to incorporate antigens for immunodiagnostic assays [7].

Searching for antigens that are expressed at different times during parasite development in the definitive host would be useful for detecting antibodies against distinct stages of the parasite. Kunitz-type (KT) protease inhibitors are low molecular weight proteins with serine protease inhibitor activity [11]. They have recently been described as involved in the regulation of major parasite and/or host-secreted cathepsin L-like cysteine proteases [12].

Among the seven genes of the Kunitz family, the *FhKT1* group (comprising *FhKT1.1, FhKT1.2*, and *FhKT1.3*) is highly expressed by the parasite and shows temporal regulation, with significantly higher levels of transcription at 24 hours after encystment, and also within mature adult parasites. The *FhKT4* gene exhibits a high level of transcription within metacercariae and newly excysted juvenile (NEJ) stages. However, unlike the *FhKT1* group, this gene is not expressed in liver-migrating immature or adult parasites [12].

In the present study, antigens belonging to the family of FhKT inhibitors (FhKT1.1, FhKT1.3 and FhKT4) were produced as fusion proteins fused to a synthetic polypeptide, using a strategy and protocol to favour the correct formation of disulphide bonds internal to the selected antigens, with a structure similar to that which they naturally possess.

This work evaluated a diagnostic assay for fasciolosis using a number of samples collected from sheep experimentally infected six weeks after infection, used in a vaccine challenge trial. The sensitivity and specificity of the ELISA indicated its usefulness for the early detection of *F. hepatica.*

## 2. Materials and methods

### 2.1. Experimental infections of sheep

Nineteen 5-month to 1 year-old sheep (male *n* = 11; female *n* = 8), creole biotype, provided by the Pampa de Olaen Cooperative, were distributed into four groups: negative control (healthy animals without parasites), orally infected with 100 *F. hepatica* metacercariae for a vaccine challenge trial (provided by Dr. Cesar Pruzzo, School of Veterinary Sciences, National University of La Plata) and naturally coccidia- or nematode-infected sheep. Animals were randomly assigned to each group and identified using numbered ear tags. Blood samples were collected by jugular vein puncture from each group at 6 weeks post *F. hepatica* infection (wpi); Negative control, Coccidia-infected, and Nematode-infected sheep. A new plastic bag was used in each animal to obtain rectal stool samples, and the presence of Coccidia: *Eimeria sp*. oocysts, and *F. hepatica* and Nematoda: *Trichuris sp*., *Trichostrongylus sp*., *Nematodirus sp*. eggs, was determined using flotation techniques (Table 1).

**Table 1.** Distribution of sheep into experimental groups.

*Groups*	Negative control	Fasciola infected	Nematoda infected	Coccidia infected
N Sex	3 2M, 1F	6 4M, 2F	5 3M, 2F	5 2M, 3F

Negative control: healthy sheep without parasites, *Fasciola* infected: sheep experimentally infected, Nematoda infected: sheep naturally infected, Coccidia infected; sheep naturally infected. M: male, F: female, N: number of animals for group

### 2.2. Ethics statements

All animal experiments were approved by the Committee for Animal Care and Use of the Agricultural Sciences School of the Catholic University of Córdoba under Resolution (21/002) in strict accordance with the recommendations of the Manual on the Care and Use of Laboratory Animals of the Canadian Council on Animal Care (CCAC).

### 2.3. Synthetic FhKT peptide

A FhKT peptide (sFhKT) was synthesized according to the sequence: IQKRCLLVEGCLGGIRSWAWDSRRGECVFVYGGCEGNDNRFDSKSSCEYNCERF described by Bozas et al., 1995 [13], ONTORES Biotechnologies (Zhejiang, China), and Genscript (New Jersey, USA). The identity and purity of the peptide was analysed by analytical reversed-phase high-performance liquid chromatography (RP-HPLC) and mass spectrometry MALDI-TOF (purity >95%).

### 2.4. Plasmid constructs

The expression plasmids used in this study were: pET26b-rFhKT 1.1, pET26b-rFhKT 1.3, and pET26b-rFhKT 4: pET26b vector carriers (Novagen; Merck KGaA; Darmstadt, Germany) with a synthetic DNA segment (GenScript) containing the corresponding FhKT (without the native or natural secretory signal peptide), the Ssp DnaX mini-intein, a CBD (chitin binding domain), and a His-tag coding sequence located downstream of the plasmid pelB signal sequence, between GCGATGGCCATG and GCGGCCGCACTC of plasmid pET26b (eliminating sequence GATATCGGAATTAATTCGGATCCGAATTCGAGCTCCGTCGACAAGCTT). The codon usage of all synthetic segments was optimized for expression in *E. coli*. The plasmids were transformed into *E.* *coli* BL21 λDE3 expression strain [14].

### 2.5. *Expression and purification of recombinant* F. hepatica *Kunitz type (FhKT1.1, FhKT1.2, FhKT4) proteins*

Transformed *E. coli* cells were grown at 37°C with shaking in 500 mL LB medium with added kanamycin (25 μg/mL) to OD600 value 0.6–0.8. Protein expression was induced by the addition of 0.4 mM IPTG, followed by incubation overnight at 20°C with shaking. Cells were then collected, resuspended in 25 ml of binding buffer (20 mMTris-HCl, pH 8.5, 500 mMNaCl, 1 mM EDTA) and lysed by high pressure homogenization (Avestin Emulsiflex C3). Supernatants of centrifuged lysates were incubated with 0.5 mL (bed volume) Ni-NTA Agarose resin (QIAGEN Hilden, Germany) at 4°C with agitation. Unbound proteins were removed by washing with 25 column bed volumes of binding buffer containing increased concentrations of imidazole (0, 20, 40, 60 mM). The His-tagged rFhKT was eluted using binding buffer containing 400 mM imidazole [15]. The purified recombinant proteins were quantified by the Bradford method using Bio-Rad Protein Assay Dye and analysed by SDS-PAGE (15%).

### 2.6. *Detection of anti-*F. hepatica *FhKT-specific antibodies in sheep sera by ELISA*

Flat-bottom 96 well micro titre half-area plates were coated in triplicate with 25 μL of 1 μg/mL of sFhKT or the recombinant antigens (rFhKT1.1, rFhKT1.3, rFhKT4) in 0.05 M carbonate buffer (pH 9.6) and incubated overnight at 4°C. After three washes with 100 μL of PBS-0.05% Tween 20 (PBST), 50 μL/well of blocking buffer,10% foetal bovine serum (FBS) (Natocor, Córdoba, Argentina) diluted in PBS was added and incubated for 1 h at 37°C. After washing three times with PBST, 25 uL of serum samples from sheep in six dilutions:2 × 10^−3^; 1 × 10^−3^ ; 5 × 10^−4^ ; 2.5 × 10^−4^ ; 1.25 × 10^−4^ ; 6.25 × 10^−5^ in PBST, 1% of FBS of PBS were added. After washing three times, 25 μL/well of HRP-conjugated donkey anti-sheep IgG (Invitrogen, Thermo Fisher Scientific, Waltham, MA, USA), diluted 1:50.000 in 1% of FBS of PBS, was added and the plates were incubated for 1 h at 37°C. Following five washes with PBST, 50 μL/well of 3,3′,5,5′, tetramethylbenzidine (TMB; Sigma Aldrich, St. Louis, MO, USA) was added and the plates were incubated at room temperature for 4 min. The reaction was stopped by the addition of 25 μL/well of 1 M sulphuric acid. Optical density was measured at 450 nm (OD450) [15].

### 2.7. Statistical analyses

A data bank was created on a Microsoft Excel 2007 spreadsheet, and was then analysed using GraphPad Prism software (version 9.0; San Diego, CA, USA) and Infostat software (version 2020; Centro de Transferencia InfoStat, FCA, UNC, Argentina; URL (http://www.infostat.com.ar). To assess the assumptions of normality, independence, and homogeneity of data variances, both graphical and formal tests were conducted. The normality of the data was verified using the Shapiro-Wilk test with a significance level of *p*-value <0.05. The Bartlett test was employed to verify the heteroscedasticity of the variances, with a significance level of *p* < 0.05. Due to the lack of homogeneity, a mixed linear model was employed for the analyses using the R programme for estimation purposes. Fisher’s LSD test was used to compare the means of optical densities obtained for sFhKT and the rFhKTs (*p*-value <0.05) at each dilution value (2 × 10^−3^; 1 × 10^−3^; 5 × 10^−4^; 2.5 × 10^−4^; 1.25 × 10^−4^; 6.25 × 10^−5^). To determine sensitivity and specificity, a contingency analysis with Fisher’s exact test was conducted, with a significance value of *p* < 0.05. The likelihood ratio was estimated based on the sensitivity and specificity values using the following formulas: +LR = sensitivity/1-specificity and -LR = 1-sensitivity/specificity [16].

The agreement between the diagnostic tests was evaluated using the kappa Cohen coefficient and interpreted according to the following scale: 0.81–1.00, excellent; 0.61–0.80, good; 0.41–0.60, moderate; 0.21–0.40, weak; and 0.0–0.20, negligible. The *kappa* index was calculated using the formula: k = (Po – Pe)/(1 –Pe), where k represents the kappa index, Po the observed agreement between the two tests (coproparasitological and ELISA), and Pe the expected agreement between the two tests. Po is calculated as the sum of true positives (TP) and true negatives (TN) divided by the total number of cases (n). Pe is determined using the formula P + N/n, where P represents the true positive results (TP) plus false positive results (FP) divided by the total number of cases (n). N is calculated as the sum of false negatives (FN) and true negatives [17,18].

## 3. Results

### 3.1. *Comparison of amino acid sequence alignment and modeling of* F. hepatica *kunitz-type inhibitors*

From the different genes coding for the Kunitz inhibitor proteins in *F. hepatica*, a distinct pattern of transcription has been described depending on the stages of the parasite in the host. Accordingly, we evaluated FhKT1.1, FhKT1.3, and FhK4, which are expressed at different times during the parasite development. Among the FhKT1 group, FhKT1.1 is most expressed during the entire cycle of the parasite, while FhKT1.3 has a late and FhKT4 an early expression. Interestingly, these proteins include different specificity as protease inhibitors. Thus, FhKT1.1 do not inhibit serine proteases, but they do inhibit cathepsin L cysteine proteases. However, FhKT1.3 inhibits cysteine and serine proteases.

**Figure 1.**
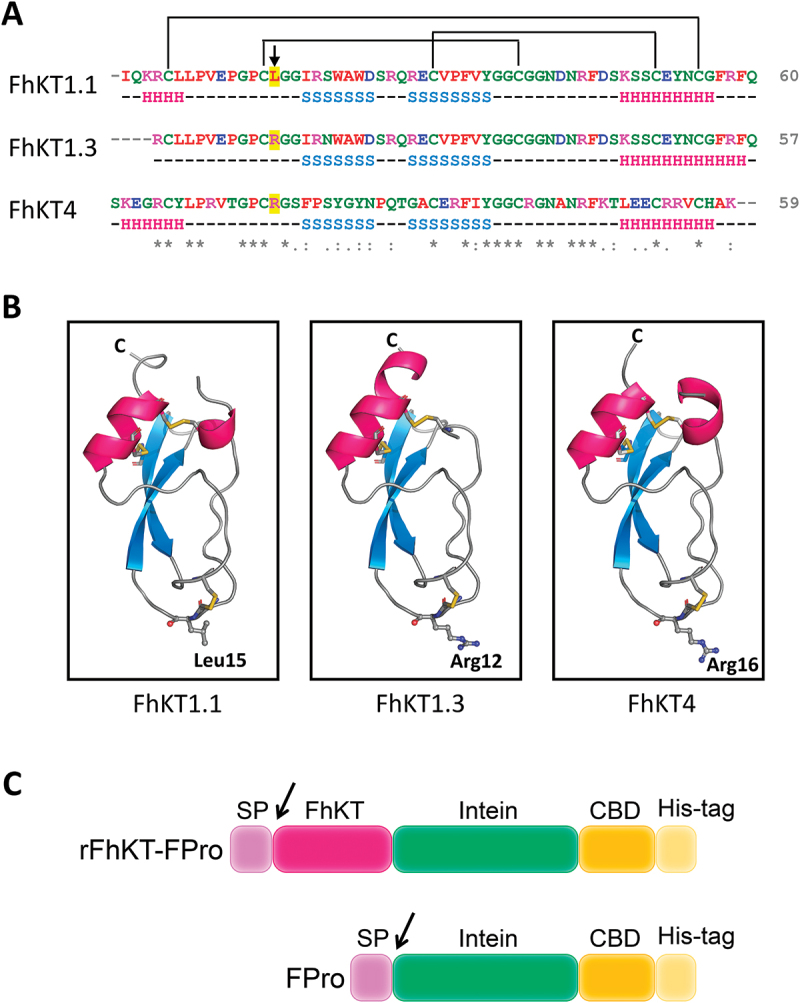
Amino acid sequence alignment and modelling of FhKT proteins (a) alignment sequence of amino acids of members belonging to the family of Kunitz type inhibitors of *F. hepatica*. Conserved amino acids are indicated by an asterisk (*), strong amino acid group similarity by a colon, and weaker group similarity by a single dot. The colour code indicates the different types of amino acids according to clustal omega. The P1 residue within the reactive site loop is indicated with an arrow. Predicted secondary structure (H = helix, S = strand) is shown below the sequences. Above the sequence, the disulphide bridge pattern is drawn. The lines connecting the cysteine residues represent the disulphide bonds. (b) ribbon representation of FhKT1.1, FhKT1.3 and FhKT4 structures predicted by AlphaFold2. P1 site residues are shown as ball-and-stick and disulphide bonds as yellow sticks. Ribbon representation of FhKT1.1, FhKT1.3 and FhKT4 secondary structures predicted by AlphaFold2. The three disulphide bonds (yellow), an alfa-helix (pink), and anti-parallel beta-sheets (blue) are shown. (c) schemes of the fusion proteins. The N-terminal pET26 pelB SP (signal peptide, 2.23 kDa), the FhKT of interest (*F. hepatica* KT-like protein, FhKT1.1: 6.79 kDa, or FhKT1.3: 6.49 kDa or FhKT4: 6.65 kDa), and the C-terminal intein (Ssp DNAx intein, 15.44 kDa), CBD (chitin binding domain, 6.61 kDa) and His-tag (poly histidine affinity tag, 1.44 kDa), are indicated. FPro corresponds to the fusion protein (intein-CBD-Histag).

We chose FhKT1.1 since FhKT1.1 and FhKT1.2 are very similar at the amino acid sequence level and are expressed similarly over time. Additionally, both have the same amino acid (Leucine) at the P1 position (Figure 1a).

Sequence alignments showed the differences in the residue at the P1 (position 15), which is related with the inhibitory profile of FhKT (Figure 1a, arrow). The predicted secondary structure displayed three disulphide bonds (yellow), an alfa-helix (pink), and anti-parallel beta-sheets (blue) (Figure 1b). A representation of the residues located at the peak of the reactive loop (Figure 1b) by Leu 15, Arg 12 and Arg 16.

We attempted to express the C-terminal His-tagged Kunitz-like proteins recombinant in the cytosol of *E. coli*, but we were unable to obtain their overexpression. Considering that FhKT proteins have three internal disulphide bridges (Figure 1a), we then tried to express them in the periplasm of *E. coli* (which is more propitious than the cytosol for the formation of disulphide bridges), for which we fused a periplasmic signal peptide at the amino end terminal of FhKT proteins and a His-tag at their carboxyl end, but we were not able to overexpress them either. Therefore, we decided to express them as fusion proteins in the periplasm of *E. coli* but fused to a larger C-terminal tag (Figure 1c), which we had already successfully used to overexpress and purify the human growth hormone, a protein that also has internal disulphide bonds. Schemes of the fusion proteins showed the SP (signal peptide), FhKT (*F. hepatica* KT-like protein), Intein (Ssp DnaX intein), CBD (chitin binding domain), His-tag (poly histidine affinity tag) and Fpro corresponding to the fusion protein (Intein-CBD-His-tag) (Figure 1c).

**Figure 2.**
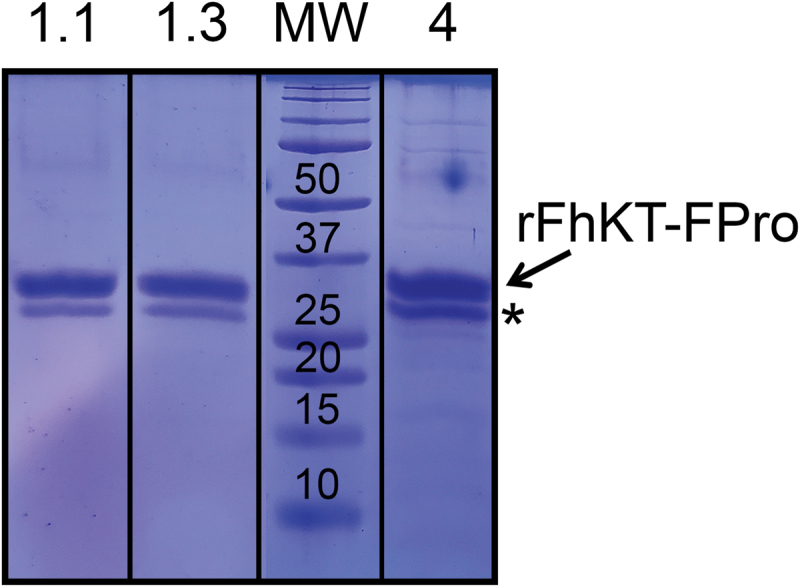
Expression and purification of rFhKt fusion proteins. A representative SDS-PAGE analysis is shown of the recombinant *E. coli*-expressed and purified fusion proteins of *F. hepatica* KT inhibitors, rFhKt1.1 (lane 1.1); rFhKt1.3 (lane 1.3), and rFhKt4 (lane 4). Lane MW: molecular weight markers. Bands with an asterisk (*) correspond to shorter derivatives of the fusion proteins rFhKT-FPro in which the DNA intein cut a small fragment of the N-terminus of the FhKT proteins during the expression-purification process.

Under experimental conditions, the rFhKTs were observed as a major protein band of approximately 30 kDa (7 kDa rFhKT monomer, 15.5 kDa DnaX, 6.6 kDa CBD and 1.4 kDa Histag-linker) (Figure 2).

### 3.2. *Assessment of anti-synthetic and rFhKt antibody responses in* F. hepatica *experimentally infected sheep*

**Figure 3.**
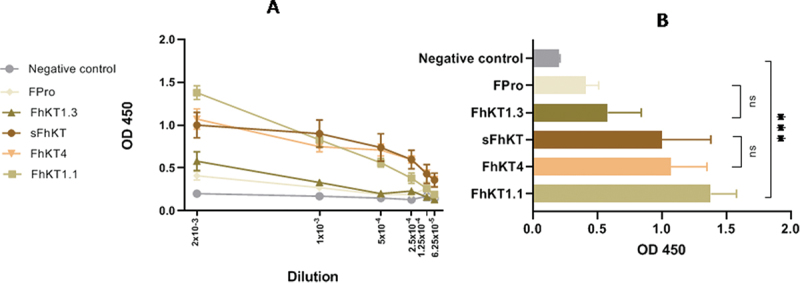
Anti-IgG antibody response in *F. hepatica*-infected sheep six weeks after experimental infection (6wpi) to sFhkt, FhKT1.1, FhKT1.3 and FhKT4. (a) mean of OD values for total IgG antibodies to sFhkt and rFhKts (*n* = 6); mean ± SEM represented at 6 serial sera dilution 2 × 10^−3^; 1 × 10^−3^; 5 × 10^−4^ ; 2.5 × 10^−4^ ; 1.25 × 10^−4^ ; 6.25 × 10^−5.^ (b) comparisons of means of OD for the 2 × 10^−3^ dilution obtained for sFhkt and the recombinants (rFhKt1.1, rFhKt1.3, rFhKt4) *** indicate statistical differences *p* < 0.001.

To test the ability of synthetic (sFhKT) and recombinant FhKT1.1, FhKT1.3 and FhKT4 to detect IgG antibody-specific responses, sera obtained from *F. hepatica* experimentally infected sheep at 6 weeksp.i. were evaluated by ELISA. Six weeks was chosen as a nearly time after *F. hepatica* infection and before the peak of IgG detection at eight weeks post infection by ELISA for sFhKT in *Fasciola*-infected sheep. The comparison of the OD using sFhKT as well as the three recombinants (FhKT1.1, FhKT1.3, and FhKT4) was determined in the serum of sheep after six weeks of infection. The antibody responses of sera from *F. hepatica*-infected and *F. hepatica*-free sheep (negative control, NC) are shown in Figure 3a. We decided to compare the OD at six dilutions of sera: 2 × 10^−3^; 1 × 10^−3^; 5 × 10^−4^; 2.5 × 10^−4^; 1.25 × 10^−4^; 6.25 × 10^−5^. Regarding infected sheep, the antibody responses to sFhKT, FhKT4 and FhKT1.1 were highest at 2 × 10¯^3^ dilution. Dilutions lower than 2 × 10¯^3^ were not useful for discriminating the OD among sera from different groups (data not shown). Decreasing OD values were obtained when the sera dilution was increased (Figure 3a). The lowest values of OD at all dilutions were obtained with fusion protein (Fpro) as well as with the sera from the *Fasciola*-free sheep (Figure 3a).

To evaluate the performance of sFhKT and the rFhKTs in the sera of *Fasciola*-infected sheep, a comparison of the means was performed at the lowest dilution, 1:500. FhKT1.1 showed the highest OD values compared to the other antigens. These differences were statistically significant (*p* < 0.001) (Figure 3b).

To assess the specificity of the IgG responses to sFhKT, FhKT1.1, FhKT1.3, and FhKT4, we compared the sera from *F. hepatica*-infected sheep with negative control individuals and those with other parasitosis. To corroborate sheep infection with other parasites, we used coproparasitological analysis to determine the presence of eggs (Nematoda) and/or oocysts (Coccidia) in the faeces of naturally infected animals (data not shown). The higher OD values corresponded to the sera from *F. hepatica*-infected sheep, except for the value obtained for FhKT1.3 (Table 2, left column).

**Table 2.** Mean values of OD for sFhkt and rFhKt from *F. hepatica* and other parasitosis-infected sheep sera.

	*F. hepatica*	Negative control	Coccidia	Nematoda
FhKTs	1.00 ± .38	0.40 ± 0.23	0.27 ± 0.1	0.39 ± 0.14
FhKT 1.1	1.24 ± .23	0.53± 0.27	0.66 ± 0.11	0.79 ± 0.33
FhKT 1.3	.58± .26	0.49 ± 0.25	0.64 ± 0.11	0.65 ± 0.20
FhKT 4	1.07 ± .28	0.81± 0.23	1.08 ± 0.47	0.82 ± 0.28

Table 2. Mean values and SD of serum OD dilution 1:500: *F.* *hepatica* n = 6, Negative control n = 5, Coccidia n = 3 and Nematoda n = 5

**Figure 4.**
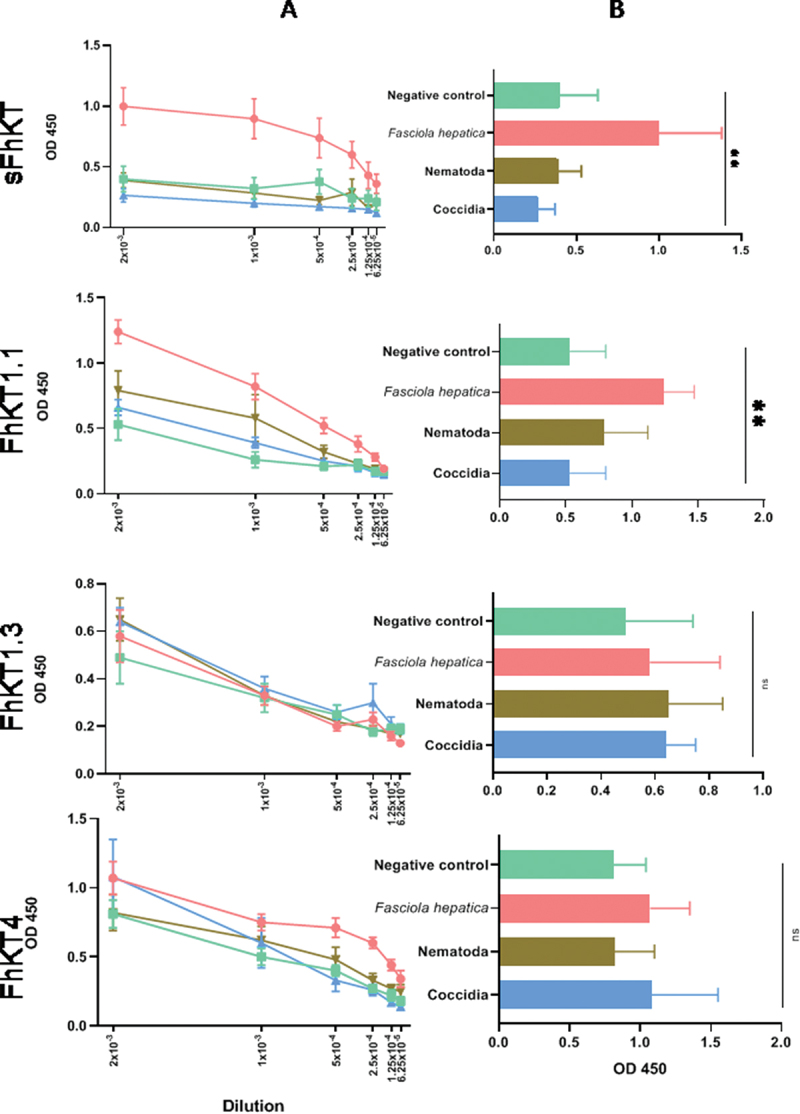
Cross-reactivity antibody responses to sFhKt, FhKT1.1, FhKT1.3, and FhKT4 from *F. hepatica*-infected sheep by ELISA compared with those infected with other parasites. (a) mean adjusted OD values at different serum dilution from *F. hepatica*-infected sheep compared to the OD values from sheep sera infected with other parasites or negative controls (*p* < 0.001). *F. hepatica n* = 6, negative control *n* = 5, Coccidia *n* = 3, and Nematoda *n* = 5. (B) differences of OD means at 2 × 10-^3^ sera dilution values using Fisher’s LSD test with a p-value <0.001.

The cross-reactivity of antibody responses to sFhKT, FhKT1.1, FhKT1.3, and FhKT4 from *F. hepatica*-infected sheep was analysed compared with those infected with other parasites. Decreasing OD values were observed when the serum dilution from all infected animals was increased (Figure 4a). However, at the first dilution(2 × 10^−3^), a greater difference was observed between the antibody response to sFhKT and FhKT1.1 of the *F. hepatica*-infected sheep compared to the OD values from sheep sera infected with other parasites or negative controls (*p* < 0.001) (Figure 4a).

We also estimated the differences in the mean OD values using a generalized mixed model with a p-value <0.001.The serum of *F. hepatica*-infected sheep showed the presence of IgG antibodies reactive to sFhKT, rFhKT1.1, and rFhKT4, with OD values statistically different from those of sheep sera infected with other parasites or negative controls (Figure 4b).

The serum of *F. hepatica*-infected sheep showed the presence of IgG antibodies reactive to sFhKT and FhKT1.1, with OD values statistically different from those of sheep sera infected with other parasites or negative controls (Figure 4b).

The precision of the diagnostic assay using FhKT was evaluated. Precision is defined as the degree of agreement between the information provided by the evaluated diagnostic test and that provided by the reference test. Diagnostic accuracy can be quantified using several metrics including sensitivity and specificity levels, likelihood ratios, Kappa index, and area under the receiver operator characteristic (ROC) curve. As seen in Table 3, the specificity and sensitivity parameters calculated by the Wilson-Brown method showed that the best combination for both parameters is obtained using FhKT1.1 (Se 0.83 and Sp 0.92), followed by sFhKT, FhKT4 and FhKT1.3.

**Table 3.** Accuracy of FhKT test diagnosis.

	Sensitivity	Specificity	+LR	-LR	*Kappa* Id	*p <*0.05
sFhKT FhKT1.1 FhKT1.3 FhKT4	0.67 0.83 0.17 0.33	1 0.92 1 0.92	NP 10.38 NP 4.1	0.33 0.18 0.83 0.73	0.80 0.91 0.28 0.49	** ** NS NS

Accuracy of FhKT test diagnosis of *F. hepatica* use of ELISA, Sensitivity; Specificity; LR: Likelihood Ratio; Kappa Id: Kappa Index; NP: not possible to calculate.

The kappa index was used to estimate the concordance between the results obtained by coproparasitological diagnosis (gold standard) and ELISA using sFhKT and the rFhKTs [17]. The highest concordance (*Kappa* index value = 0.91) was obtained for FhKT1.1, followed by sFhKT, FhKT4, and FhKT1.3 (Table 3).

We assessed the effectiveness of the diagnostic test by determining likelihood. As the positive likelihood ratio (LR+) increases, the test becomes more valuable in confirming the presence of the disease with more certainty. Similarly, a low negative likelihood ratio (LR-) enhances its efficiency in ruling out the disease. Table 3 illustrates that FhKT1.1 achieves an optimal balance, with a high LR+ value and the lowest LR- value, emphasizing its diagnostic utility.

**Figure 5.**
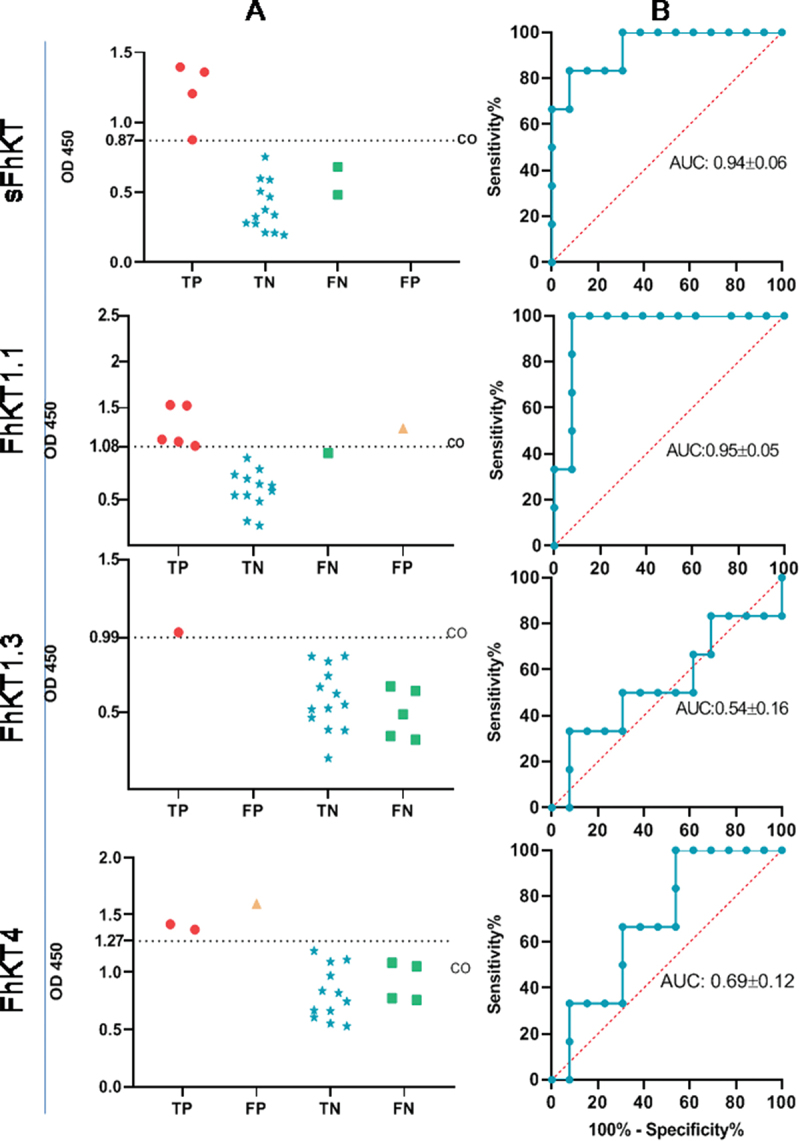
Discriminant capacity of the diagnosis of *F. hepatica* in sheep using FhKT. (a) cut-off points (CO) for each FhKT, TP: true positive; FP: false positive; TN: true negative; FN: false negative. (b) ROC curves for each FhKT, using OD at 2 × 10-^3^ dilution sera of *F. hepatica n* = 6, negative control *n* = 5, Coccidia *n* = 3 and Nematoda *n* = 5, AUC: area under the curve.

To assess the discriminatory ability of the sFhKT and the rFhKTs by ELISA, we calculated the cut-off values and ROC curve. The cut-off point between the groups for each sFhKT and rFhKT was set as the mean value plus two deviations of the negative control serum obtained with each antigen (Figure 5a). FhKT1.1 obtained the highest value for true positive (TP) results followed for sFhKT and the lowest value for false negative results (FN).

The receiver operating characteristic (ROC) curve was used to determine the diagnostic accuracy of ELISA. The area under the ROC curve (AUC), which indicates how good the test is to discriminate sheep with and without *F. hepatica* over the entire range of possible cut-off points, shows that the highest values were obtained for the ELISA using FhKT1.1 and sFhKT, respectively (Figure 5b).

**Figure 6.**
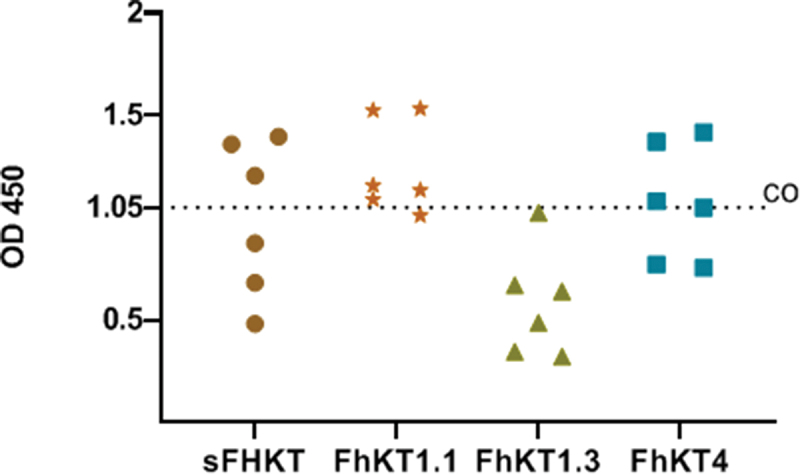
Comparison of the performance of synthetic Kunitz (sFhkt) and recombinants (FhKT1.1, FhKT1.3, FhKT4) in the diagnosis of *F. hepatica*-infected sheep. (CO) average cut-off value for each antigen (CO: 1.05 OD) obtained from individual cut-off values for each sFhkt or rFhKt (0.87; 1.08; 0.99; 1.27).

To compare the ability of sFhKT and the rFhKTs to differentiate between *F. hepatica*-infected sheep and non-infected sheep, an average cut-off value (CO: 1.05 OD) was obtained from individual cut-off values for each sFhKT or rFhKT (0.87; 1.08; 0.99; 1.27). The results obtained, as shown in (Figure 6), indicate that FhKT1.1 performed better than the others.

## 4. Discussion

Fasciolosis is a zoonotic disease that is widely distributed around the world, including Latin America, and has an economic impact on regional economies [19–21]. Specific treatment against *F. hepatica* requires an accurate diagnosis that differentiates this parasite from other helminths, fundamentally nematodes, which are the most frequent in sheep. Access to the diagnosis of fascioliasis in developing countries needs local production of antigens that are affordable for generating diagnostic kits. The immune response to the secretory-excretory products of the parasite can be used for the diagnosis of the disease [8].

*F. hepatica* is a parasite that releases copious excretory-secretory products through the tissues of mammals during its life cycle. Among these products are several enzymes from the cathepsin family that contribute to tissue digestion. The expression and secretion of proteases, such as cathepsin L and B, are essential for the digestion of both the intestinal wall and the liver tissue by the parasite until settlement in the bile ducts [22]. To resist the action of these proteases, it has been postulated that Kunitz family proteins are capable of inhibiting the action of both cysteine and serine proteases secreted by the host and/or parasite [12,23].

Kunitz-type proteins are a group of protease inhibitors that are ubiquitous, found in arthropods, helminths, and mammals. The inhibitory specificity of the Kunitz domain varies with the particular amino acids at the reactive sites and their function depends on the organism [24,25]. In vertebrates, Kunitz inhibitors play a major role in inflammatory processes while, in invertebrates such as scorpions, cone snails are involved in both neurotoxic and protease inhibitory activity and, in blood-sucking arthropods, they function as anti-coagulant factors [26–28].

In the case of helminths like *F. hepatica*, these inhibitors with cysteine protease inhibitory activity are expressed as a temporally regulated family of members to control the activity of cysteine proteases during migration, growth, and development. Furthermore, they play a significant role in inhibiting key host lysosomal cathepsin L involved in antigen processing, which may serve to modulate host responses to parasite molecules in the context of the parasite *F. hepatica* infection [12].

Apart from this unique property, these proteins were copiously secreted by the parasite early and throughout its evolution in the host [11]. Their ability to induce antibodies has also been demonstrated in mice immunized with sFhKT [29] and sheep. However, these proteins have not yet been evaluated in the diagnostic tests for fasciolosis.

The objective of this study was to compare the performance of three recombinant proteins (rFhKT1.1, FhKT1.3 and FhKT4) in a diagnostic ELISA for fasciolosis in sheep. The three Kunitz-type inhibitor molecules were expressed and purified as a recombinant fusion protein in *E. coli*, based on a protocol previously described for the production of recombinant human growth hormone (rhGH) [14]. The amount of recombinant protein was sufficient to perform the coating in the ELISA. The significance of achieving good protein recovery is underscored by the preference for recombinant antigens over native antigens in commercial immunodiagnostic tests [30]. This preference is based on the ability to produce recombinant antigens consistently and extensively, facilitating large-scale manufacturing. By utilizing recombinant antigens, a stable and reliable supply is ensured, which is important in maintaining the overall quality and reproducibility of diagnostic assays.

Moreover, solid-phase synthesis, as a production method for Kunitz peptide, is known to be expensive and often inefficient in yielding specific quantities. Additionally, it may not be readily accessible for production in countries like Argentina. The latter is particularly relevant because fasciolosis in sheep is likely to escalate and spread in the coming years due to climate change, with temperature increases that favour the persistence of the parasite and its intermediate hosts [31].

Based on the ELISA results, it is evident that FhKT1.1 was superior to the other recombinant proteins used. This is not surprising asFhKT1.1 is highly expressed, particularly within the early infective stages of the parasite. Interestingly, although rFhKT1.3, a part of the FhKT1 clade, was not effective for the detection of antibodies by ELISA in experimentally infected sheep, this cannot be attributed to the lower gene expression of FhKY1.3, as analysis using RNA-Seq data revealed that all three members of the FhKT1 clade (FhKT1.1, 1.2, and 1.3) were highly expressed at all stages within the mammalian host [11]. Additionally, proteomic analysis of NEJ adult worm secretions (ES products) and EVs identified peptides that corresponded to proteins derived from the gene products of FhKT1.1, 1.2, and 1.3. Interestingly, sequence alignment analysis of FhKT1.3 reveals that the P1 position (position 15), located within the reactive loop of KT inhibitors and critical for binding to and inhibiting the target protease, is occupied by a positively charged arginine, in contrast to the hydrophobic leucine residues found in FhKT1.1 and FhKT1.2 [23].

Whether the presence of leucine at P1 plays a role in antibody recognition against FhKT is unknown; however, FhKT4 has arginine at P1 and performed better than FhKT1.3 as an antigen in ELISA, suggesting the irrelevance of the residue present at P1 in FhKT as a target antigen in ELISA for the serological diagnosis of fasciolosis in sheep.

It is important to highlight that comparing the different FhKTs as target antigens in ELISA using diagnostic precision variables enabled us to conclude that only FhKT1.1 identified all the experimentally infected individuals, outperforming the others [16–18,32,33]

The structure of the amino acid sequence would affect its ability to cleave different proteases, but we do not know whether this influences its ability to be recognized by antibodies. However, the differences observed in the secondary and tertiary structures may explain the differential behaviour in the recognition of antibodies, according to what was observed in the ELISA results.

Regarding cross-reactivity, it was found that FhKT1.1 performed best when challenged with sera from sheep infected with coccidia or nematodes, parasites of high frequency in flocks; however, it is recommended to challenge with a greater variety of sera, including sheep infected with flatworms, to provide a more complete understanding of its specificity and potential application in diagnostic tests or immunity studies against *F. hepatica* in sheep [34].

Additionally, comparing OD values using a contingency analysis that allows assessment of the performance of all target antigens in ELISA demonstrates that FhKT1.1 is the most efficient in identifying positive individuals, while sFhKT identifies 50% of these. This data together validates the use of FhKT1.1 for the serodiagnosis of fasciolosis in sheep in the early stages of infection, prior to detection of the disease by coproparasitological methods.

Finally, these results may enable the development of an efficient and available diagnostic test for sheep farmers, the rational use of antiparasitic drugs, and the lower environmental impact of their waste.

## Data Availability

The datasets generated and/or analysed during the current study are available in the Mendeley Data repository V1, doi: 10.17632/zkc7jdjn83.1
